# Language impairment in Parkinson’s disease: fMRI study of sentence reading comprehension

**DOI:** 10.3389/fnagi.2023.1117473

**Published:** 2023-03-09

**Authors:** Lubomira Novakova, Martin Gajdos, Jana Markova, Alice Martinkovicova, Zuzana Kosutzka, Jana Svantnerova, Peter Valkovic, Zsolt Csefalvay, Irena Rektorova

**Affiliations:** ^1^Brain and Mind Research, Central European Institute of Technology, Masaryk University, Brno, Czechia; ^2^Department of Communication Disorders, Faculty of Education, Comenius University, Bratislava, Slovakia; ^3^Second Department of Neurology, Faculty of Medicine, Comenius University, Bratislava, Slovakia

**Keywords:** Parkinson’s disease, language impairment, sentence reading comprehension, task fMRI, functional connectivity, striatum

## Abstract

Parkinson’s disease (PD) affects the language processes, with a significant impact on the patients’ daily communication. We aimed to describe specific alterations in the comprehension of syntactically complex sentences in patients with PD (PwPD) as compared to healthy controls (HC) and to identify the neural underpinnings of these deficits using a functional connectivity analysis of the striatum. A total of 20 patients PwPD and 15 HC participated in the fMRI study. We analyzed their performance of a Test of sentence comprehension (ToSC) adjusted for fMRI. A task-dependent functional connectivity analysis of the striatum was conducted using the psychophysiological interaction method (PPI). On the behavioral level, the PwPD scored significantly lower (mean ± sd: 77.3 ± 12.6) in the total ToSC score than the HC did (mean ± sd: 86.6 ± 8.0), *p* = 0.02, and the difference was also significant specifically for sentences with a non-canonical word order (PD-mean ± sd: 69.9 ± 14.1, HC-mean ± sd: 80.2 ± 11.5, *p* = 0.04). Using PPI, we found a statistically significant difference between the PwPD and the HC in connectivity from the right striatum to the supplementary motor area [SMA, (4 8 53)] for non-canonical sentences. This PPI connectivity was negatively correlated with the ToSC accuracy of non-canonical sentences in the PwPD. Our results showed disturbed sentence reading comprehension in the PwPD with altered task-dependent functional connectivity from the right striatum to the SMA, which supports the synchronization of the temporal and sequential aspects of language processing. The study revealed that subcortical-cortical networks (striatal-frontal loop) in PwPD are compromised, leading to impaired comprehension of syntactically complex sentences.

## Introduction

The hallmark of Parkinson’s disease (PD) is the degeneration of dopaminergic neurons in the basal ganglia; it affects the motor system and may also affect language processes. Most of the attention in the field of communication disorders in Parkinson’s disease (PD) is paid to motor speech changes. Hypokinetic dysarthria (decreased variations in pitch and loudness and imprecise articulation) can be found using advanced analytic techniques on speech recordings, and non-invasive brain stimulation can be used as a modifying therapy for it (Brabenec et al., 2019, 2021; Klobusiakova et al., 2021).

Apart from the difficulties in speech, language problems also occur in patients with PD (PwPD). Language is one of the main cognitive domains and language performance might be influenced by overall cognitive functioning. Executive and visuospatial domain is typically impaired in early stages of cognitive decline in PD (Aarsland et al., 2021), but other domains including language might be impaired as well.

In the language production component there are deficits in grammatical formation, syntactical complexity, content/informativeness of narrative speech, specific problems with words representing actions (action naming/fluency)/manipulable objects, and non-verbal communication (Smith and Caplan, 2018). Recent study (Lowit et al., 2022) showed higher rate of grammatical and lexical errors in sentence production tasks in PwPD and even though the authors found the link between executive function and language performance in grammar tasks but performance in Sentence Generation and Narrative was independent of it. Two fMRI studies focused on brain activation in PD during action naming and processing found that PwPD seemed to process action verbs *via* the non-motor cortical networks (Péran et al., 2009; Abrevaya et al., 2016). In the language comprehension component, processing complex grammatical and syntactical structures, and narrative comprehension seem to be deficient in PwPD (Ash et al., 2012; Gross et al., 2012, 2013; Grossman et al., 2012, 2017). PwPD, as compared to healthy controls (HC), were impaired in syntactic comprehension and judgment, and at naming manipulated objects but not at naming non-manipulated objects (Johari et al., 2019).

Most authors attribute the sentence comprehension deficits in PD to limited cognitive resources (Grossman et al., 2000) and problems mostly in the executive domain, such as difficulties in set-shifting, attention, inhibition, and working memory (Grossman et al., 2002; Lee et al., 2003; Hochstadt et al., 2006; Hochstadt, 2009; Colman et al., 2011; Marková et al., 2018). Angwin et al. (2005) reported deficits in lexical-semantic processing during sentence comprehension. Their results suggest that some sentence comprehension deficits in PD may be related to a reduction in information processing speed. By contrast, Skeel et al. (2001) found deficits in processing both semantically and syntactically loaded sentences in PD, but no deficits in working memory and no significant impact of dopaminergic medication. Some authors also found that linguistic impairments might be independent of cognitive deficits, such as in executive functioning (Troche and Altmann, 2012) or link between executive function and language performance might be task dependent (Lowit et al., 2022).

Comprehension of non-canonical, passive, or object-relative structures seems to cause the most problems in PD (Lee et al., 2003; Angwin et al., 2005). Non-canonical order refers to sentences in which the *patient* (affected by the action or object) precedes the *agent* of the action (subject). In English, a rigid word order encodes the grammatical relations between words (if the word order of a sentence–e.g., The mother is kissing the daughter–is changed, the meaning of the sentence also changes: The daughter is kissing the mother). Non-canonical order in English is accomplished mostly by using passive structures (The mother is kissed by the daughter). By contrast, languages such as Slovak or Czech have a more flexible word order and these grammatical relations are indicated by case markings on the nouns (nominative–NOM vs. accusative–ACC). An example of a sentence with a canonical order in Slovak: Dcéra (NOM) bozkáva mamu (ACC), meaning “The daughter is kissing the mother”; and with non-canonical order: Mamu (ACC) bozkáva dcéra (NOM). This type of non-canonical word order sentence does not have the same function in English; the meaning is: “The mother is kissed by the daughter” but it is not passive in Slovak. Both sentences are active and typical for Slovak language but canonical word order has a processing advantage over non-canonical word order (for more details see Supplementary Material or Marková et al., 2017).

The neural network for sentence comprehension in healthy subjects (Walenski et al., 2019), calculated using activation likelihood estimation based meta-analyses, showed a significant involvement of regions such as the inferior frontal gyrus, insula, temporal pole, precentral gyrus, supplementary motor area (SMA), superior and middle temporal gyrus, angular gyrus, and temporal occipital fusiform cortex. The results from an analysis of non-canonical sentences showed the engagement of the frontal and posterior temporal regions as well as the left angular gyrus and the right insula.

Few neuroimaging studies using fMRI have been concentrated on language comprehension in PD. Grossman et al. (2003) found that impaired sentence comprehension in PD was associated with changes in a large-scale network important for cognitive resources during sentence processing (decreased activation mainly of the striatum and increased compensatory activation of structures associated with working memory). In accordance with these results, another group found that dysfunction of the caudate nucleus networks underlies PwPD’ difficulty in dealing with complex sentence structures (before vs. after sentences) as measured in fMRI (Ye et al., 2012).

Although sentence comprehension was shown to be compromised in PD, the pathogenic role of abnormalities affecting functional integration between large-scale brain networks has not been demonstrated conclusively and a limited number of studies have focused on disturbed sentence comprehension using fMRI. We specifically focused on the striatum, because the loss of dopaminergic neurons and their projections to the striatum produces the core motor symptoms of PD and contributes to some of the cognitive and behavioral features, such as dysfunction of the basal ganglia thalamocortical network (McGregor and Nelson, 2019). We studied the striatal connectivity because previous research hinted that its alterations were connected with language impairment in PD (Grossman et al., 2003; Péran et al., 2009; Ye et al., 2012; Abrevaya et al., 2016). Our aim was to describe specific alterations in the comprehension of syntactically complex sentences in PD as compared to HC and to identify the neural underpinnings of these deficits using a functional connectivity analysis of the striatum. Our hypothesis was that PwPD show deficits in their comprehension of syntactically complex sentences (especially of non-canonical structures) with altered task-dependent functional connectivity from the striatum as compared to HC.

## Methods

### Participants and examination protocol

We included clinically diagnosed non-demented PwPD (Postuma et al., 2015) and HC with the exclusion criteria of serious brain injury, major psychiatric disorder or central nervous system disease (other than PD in PwPD group), DBS or pump-delivered therapy, alcohol/drug abuse, and any contraindications for MRI. To evaluate cognitive functions, we used the following neuropsychological tests (as described in Marková et al. (2018)], in which we also indicate the cognitive domain that they primarily measure: Montreal Cognitive Assessment (MOCA)–general cognitive functioning [a cut-off score for a normal performance and inclusion in our protocol was ≥24 points as suggested by Lucza et al. (2015)], Rey’s figure–visuo-spatial functions, visual memory; repetition of numbers and letters–verbal working memory and working memory capacity; Stroop test–processing speed, sensitivity to interference, cognitive flexibility; Trail Making Test–processing speed (Part A), cognitive flexibility and task switching (Part B); semantic and phonemic verbal fluency – initiation, language skills, verbal executive; auditory verbal learning test (AVLT)—memory learning test – immediate and delayed verbal memory. The neurocognitive battery was administered only in the group of PwPD. Beck Depression Inventory-II (BDI-II) was used as a subjective measure of depressive symptoms (no cut-off score was used, which is mentioned as a limitation of our study in the Section“Discussion”).

Our cohort consisted of 20 PwPD (age 60.4 ± 7.6 years; 60% men; disease duration: 5.5 ± 3.1 years; The Unified Parkinson’s Disease Rating Scale–UPDRS III 27.8 ± 10.2; Hoehn and Yahr–H and Y score 2 for all subjects except one, who had 2.5; Levodopa Equivalent Daily Dose–LEDD 1,156 ± 603.1 mg/day; 10 patients had right-sided PD symptom predominance, 8 had left-sided symptom predominance and 2 were without predominance; MOCA 27.4 ± 2.1; BDI-II 13.6 ± 8.6) and 15 HC (age 64.5 ± 4.3 years; 40% men; MOCA 27.9 ± 1.5; BDI-II 4.0 ± 2.3). The groups did not significantly differ in age, gender, or education, but they differed in BDI-II (*p* = 0.0002).

### Experimental design–the test of sentence comprehension

**FIGURE 1:**
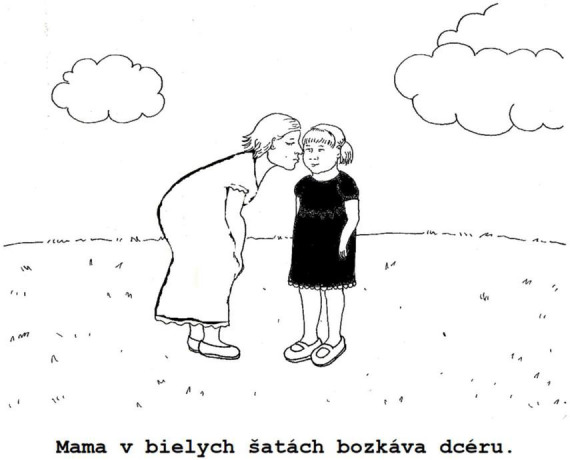
Test of sentence comprehension, sample picture, English translation: The mother in a white dress is kissing the daughter.

All participants were right-handed Slovak native speakers and we analyzed the performance of a language-specific Slovak version of the test of sentence comprehension (ToSC) adjusted for fMRI (Figure 1) (for more details Marková et al., 2018, 2017). The ToSC consists of black-and-white pictures depicting an action (the roles of the main participants may reverse and they differ visually from each other, e.g., in the shade of their clothes) and semantically reversible sentences in which the main participants (subjects and objects) can exchange their thematic roles and the sentence still remains meaningful. These syntactically complex sentences contain transitive verbs and two nouns (person/animal), one functioning as a subject and the other as an object, both of which can perform the action expressed by the verb. In sentences with a “canonical order” of thematic roles, the first noun in the sentence is assigned the role of an agent (i.e., the doer of an action); in sentences with a non-canonical order of thematic roles, the first noun in the sentence plays the role of the patient (i.e., the entity that undergoes or is affected by some action). Participants quietly read the sentence below the picture on the screen during fMRI and their task was to decide whether the sentence matched the scene in the picture by pressing a YES or NO button. Figure 1 shows an example of a picture that was presented together with the sentence “The mother (ACC) is kissing the daughter (NOM) in the white dress.” All tested sentences were active and all of them were unambiguous because of the morphological cue at nouns and pronouns (for more details see Supplementary Material or Marková et al., 2017). The control task used the same picture material; there was a syntactically simple sentence below “The scene is taking place indoors.” Or the scene is taking place outdoors.’ and the participants had to decide whether decide whether the sentence matched the scene by pressing a YES or NO button. All participants were properly instructed and practiced the task before they were scanned. Only participants who understood the instructions were eligible for the study. All patients were tested in their best ON state in the morning i.e., patients took their regular morning dopaminergic medication 1 h before the data acquisition. They had to be without any changes in dopaminergic treatment in the last 3 months. All subjects signed an informed consent form that was approved by the local ethics committee.

### MRI examination

3T Siemens Prisma MR scanner (Siemens Corp., Erlangen, Germany) was used for data acquisition at the Central European Institute of Technology (CEITEC), Masaryk University in Brno, using the T1 MPRAGE sequence (TR 2,300 ms; TE 2.33 ms; voxel size 1 × 1 × 1 mm; FoV 224 × 224 mm; flip angle 8°; 240 sagittal slices) and multiecho BOLD fMRI sequence for two sessions of the task fMRI [TR 987 ms; TE (14.2, 35.4) ms; voxel size 2.561 × 2.561 × 2.5 mm; FoV 210 mm; flip angle 45°; 64 axial slices; 500 scans per session; multiband factor 4]. The fMRI task consisted of 96 trials, divided into 2 sessions (48 trials each). During the trial (10 s), a picture was presented with a sentence describing the picture (until the participant responded by pressing a YES or NO button, up to 5 s), followed by a screen with a fixation cross (minimally 5 s). The duration time of the whole fMRI task (2 sessions together) was 16 min. The sentences were characterized by the condition of canonicity (canonical, non-canonical, and control; 32 trials each). The rate of correct and false statements was 1:1 (48 to 48). Conditions were randomly intermixed and the number of correct responses (accuracy of ToSC) was an outcome measure.

### Data analyses

The fMRI data were preprocessed using the SPM 12 toolbox running under Matlab 2019a (MathWorks, Inc.). Preprocessing included realignment, multiecho merging based on CNR (Poser et al., 2006), spatial normalization, and smoothing (5 mm FWHM Gaussian filter). We excluded subjects for whom the framewise displacement metric exceeded 0.5 mm in more than 20% of the scans in each of the sessions. For fMRI analyses, four PwPD were excluded from analysis due to excessive movements. We controlled data for spatial abnormalities (e.g., dropouts) with the Mask Explorer tool (Gajdoš et al., 2016). We prepared masks of the striatum based on the structural anatomical striatal atlas (Tziortzi et al., 2011) available within the FSL software package. This atlas is subdivided into three striatal subregions–caudate, putamen, and ventral striatum. We prepared masks of the left and right striatum consisting of all three subregions present in the left and right hemispheres.

The Mann–Whitney U-test was used to calculate the differences in accuracy between groups. Spearman correlation coefficients were calculated between behavioral outcomes (accuracy of the ToSC fMRI task) and neuropsychological scores in patients. We used general linear model (GLM) to filter out the effect of those neuropsychological measures that at least moderately correlated with our behavioral outcomes (accuracy of the ToSC fMRI task) to find out how much language domain (in our case comprehension of syntactically complex sentences) might be independently impaired of other cognitive domains in PD.

A task-dependent functional connectivity analysis from the striatum was studied using the psychophysiological interaction method (PPI) (O’Reilly et al., 2012). We used whole-brain seed PPI computed with GLM. We modeled regressors for the activity of the seed (physiological regressor), for the task conditions (psychological regressors: canonical, non-canonical and control condition convolved with hemodynamic response function) and interaction of physiological and psychological regressors (PPI regressors). The model also involved 24 confound regressors for movement parameters (translations and rotations, their squares, their differences, and squares of their differences), 2 confound regressors with signals originating in white matter and cerebrospinal fluid and confound regressors for scans exceeding 1.5 mm of framewise displacement. All reported results had a statistical significance threshold set to *p* < 0.05 with FWE correction at the cluster level with initial cut *p* = 0.005 uncorrected. All reported coordinates are in MNI space. Spearman partial correlations, controlling for the effects of age and the BDI-II were calculated between the PPI parameters of interest (i.e., with significant differences between both groups) and behavioral outcomes (accuracy of the ToSC fMRI task in non-canonical sentences). Only medium to strong correlations with | r| > 0.4 were reported.

## Results

On the behavioral level, the PwPD scored significantly lower (mean ± sd: 77.3 ± 12.6) on total score of ToSC fMRI task than HC (mean ± sd: 86.6 ± 8.0), *p* = 0.02, and the difference was significant for both canonical (PD-mean ± sd: 84.6 ± 12.7, HC-mean ± sd: 92.9 ± 5.8, *p* = 0.03) and non-canonical sentences (PD-mean ± sd: 69.9 ± 14.1, HC-mean ± sd: 80.2 ± 11.5, *p* = 0.04). Compared to a control task, the difference between PwPD and HC in comprehending syntactically complex sentences nearly reached the level of significance (*p* = 0.05). In PwPD, the overall accuracy positively correlated with phonemic fluency [*r*(14) = 0.49, *p* = 0.03] and the accuracy of non-canonical sentences positively correlated with both phonemic fluency [*r*(14) = 0.40, *p* = 0.08] and interference of Stroop task [*r*(14) = 0.43, *p* = 0.06], but *p*-values were non-significant, for more details see Supplementary Table 1 in the Supplementary Material. After filtering the PwPD data from the effect of these two neuropsychological measures that correlated with the accuracy (phonemic fluency and interference of Stroop task) the differences between groups in accuracy was even more prominent in both total score of ToSC fMRI task (*p* = 0.01) and specifically for non-canonical sentences (*p* = 0.03).

**FIGURE 2:**
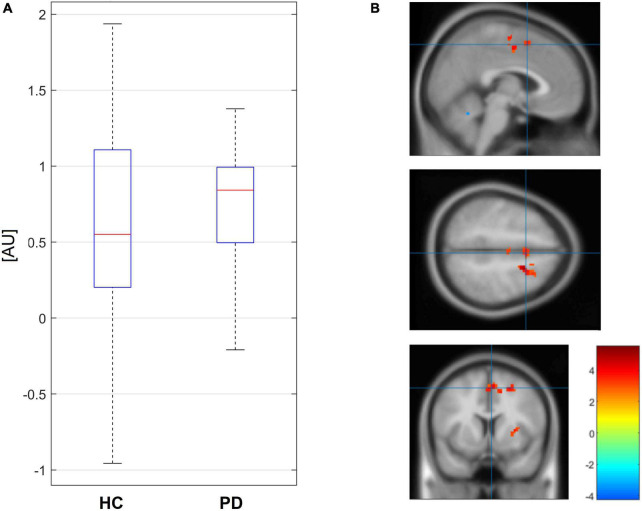
Boxplots of values of seed PPI connectivity in contrast for non-canonical stimuli from right striatum to SMA (3.73, 8.37, and 52.5) are depicted on the left **(A)**. Localization of the position in parametric map of the two sample t-test between PD and HC (three orthogonal views) with initial cutoff *p* = 0.005 for cluster level inference is on the right **(B)**.

**FIGURE 3:**
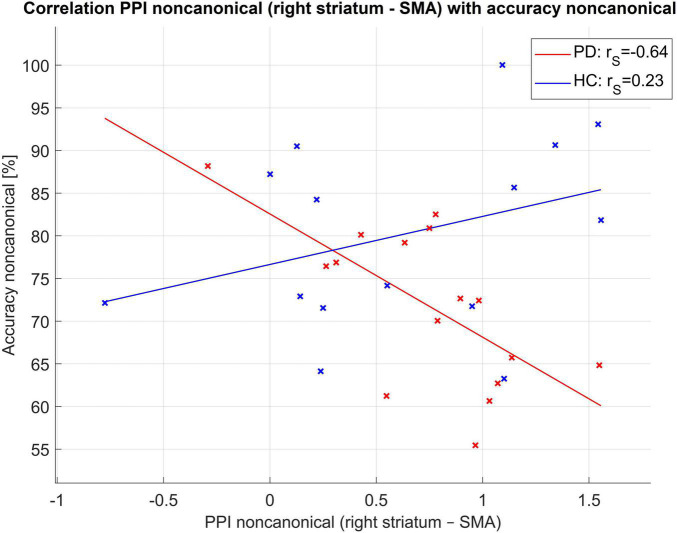
Correlation of PPI connectivity seed striatum right to SMA and accuracy of non-canonical sentences. Here r_S_ for Spearman correlation coefficient.

Using PPI, we found that there was a statistically significant difference between PD and HC in connectivity from the right striatum to the SMA [4 8 53] for non-canonical sentences (the connectivity was higher in PD than in HC), Figure 2, for more details on PPI connectivity see Supplementary Table 2 and Supplementary Figures 1–4 in the Supplementary Material. This PPI connectivity was negatively correlated with the accuracy of the comprehension of non-canonical sentences in PwPD [*r*(14) = −0.64, *p* = 0.01], see Figure 3.

## Discussion

Primary findings of this study showed worse performance of sentence comprehension in PwPD as compared to HC. A significant increase of functional connectivity between the right striatum and the SMA was also found in PwPD, which was negatively correlated with their impaired accuracy of non-canonical sentence comprehension. These findings suggest a compromised striatal-frontal loop in PwPD that may be related to their diminished comprehension of syntactically complex sentences.

Behavioral results that revealed problems in the PwPD with comprehension of syntactically complex sentences are in line with previous research (Lee et al., 2003; Angwin et al., 2005). Marková et al. (2018), using the same test material (ToSC) as was adapted in our fMRI task, showed that PwPD were significantly worse than HC in decoding sentences with non-canonical order. In other words, we replicated this behavioral outcome in a different sample under different data collection conditions (reading/visual sentence comprehension inside of MRI as compared to auditory sentence comprehension in ambulance settings). In our study all patients were tested in their best ON medication state which might influence their performance in sentence comprehension task. Previously it was suggested that dopamine supports sentence comprehension in PD and that PwPD OFF levodopa have even poorer comprehension of syntactically complex sentences (Grossman et al., 2001) and slower lexical activation (Angwin et al., 2007) as compared to ON state. Studying a different neurodegenerative disease group and using ToSC, Marková et al. (2017) showed that patients with even mild Alzheimer’s disease (AD) have more serious problems processing sentences with a non-canonical order.

Our results indicated that overall accuracy in an fMRI task and accuracy for non-canonical sentences correlated mostly with executive functions (in this study related to phonemic fluency, interference of Stroop task), such as difficulties in set-shifting, mental search and monitoring, and inhibition. Similarly, executive functioning was previously described as being associated with impaired language comprehension difficulties in PD (Grossman et al., 2002; Lee et al., 2003; Hochstadt et al., 2006; Hochstadt, 2009; Colman et al., 2011; Marková et al., 2018). After filtering out the effect of executive functions, the differences between groups did not disappear; instead, they were even slightly more prominent. Thus, impaired executive functioning does not represent the main cause of the problems with comprehension of syntactically complex sentences in PD. The fact that we did not find the link between executive functioning and language deficits might be task-dependent as was shown previously (Lowit et al., 2022), in our study related to choice of phonemic fluency, interference of Stroop task and sentence reading comprehension task. Alternatively we might as other authors (Troche and Altmann, 2012) also speculate that language domain (in our case comprehension of syntactically complex sentences) might be independently impaired of other cognitive domains (e.g., executive functions). For the sentence comprehension in Slovak it is important to perceive and recognize the morphological cue and to suppress the strategy “the first noun in a sentence is the agent.” The reason of worse performance of PwPD in non-canonical sentences might be the “insensitivity” to a morphological cue, or the insufficient sources for its processing.

In our research we aimed to describe subcortical-cortical effective functional connectivity differences in PwPD compared to HC while performing a sentence comprehension task. We found an increase of connectivity between the right striatum and the SMA in the PwPD group, but this increase was not associated with a better performance; instead, we found a negative correlation with task accuracy. The SMA is connected with the basal ganglia (striatum) circuit that supports the synchronization of temporal and sequential aspects (i.e., syntax) in language processing (syntactic processes under attentional control) (Kotz et al., 2009). The SMA has many other roles in PD with regard to motor aspects; it has also been shown to be a part of the neural network for sentence comprehension in healthy subjects (Walenski et al., 2019).

Maladaptation in an effort to compensate in neurodegenerative disorders has been described previously (Cramer et al., 2011). Neuroplasticity is a life-long process that intermediates the structural and functional response of dendrites, axons, and synapses to new experience or some pathological changes (Mesulam, 1999). It was described previously in mild cognitive impairment preceding AD that there is a phase of increased hippocampal activation early in the course of the disease followed by a subsequent decrease as the disease progresses (Dickerson et al., 2005). Similarly, in PD it was shown that the decline in heightened plasticity that was present at onset of the disease may reflect failure of compensatory mechanisms that maintained function in the preclinical state (Kojovic et al., 2015). In degenerative diseases with increasing pathology over time causes compensatory mechanisms to fail or become pathogenic in their effects on vulnerable neuronal populations, thereby destabilizing networks (Mesulam, 1999).

The limitations of this study are relatively small sample size, examining in the ON medication state, the lack of neuropsychological evaluation of HC and no cut-off score in BDI-II applied. In sum, we believe that subcortical-cortical networks (especially striatal-frontal loop) in PwPD are compromised, leading to deficits in comprehension of syntactically complex sentences. In the future, more studies are needed to explore the functional and structural (Hope et al., 2019; Sejnoha Minsterova et al., 2020) connectivity alterations that may be responsible for the language impairment in PD and the therapeutical approaches to address these language dysfunctions. Non-invasive brain stimulation techniques that showed potential to be modifying therapy for disturbed speech in PwPD (Brabenec et al., 2019, 2021) might also explain the causality of the altered connectivity of distinct cortical regions with the basal ganglia on specific language task performance and might modify task-related connectivity for improved language task performance (Novakova et al., 2020). But this approach for language impairment in PD needs further research and validation.

## Data availability statement

The raw data supporting the conclusions of this article will be made available by the authors, without undue reservation.

## Ethics statement

The studies involving human participants were reviewed and approved by the Ethics Committee of the University Hospital Bratislava. The patients/participants provided their written informed consent to participate in this study.

## Author contributions

LN, IR, ZC, PV, and JM: study conception and design. AM, ZK, and JS: data collection. LN and MG: data analysis. LN, MG, PV, ZC, and IR: interpretation of results. LN: manuscript preparation. All authors reviewed the results and approved the final version of the manuscript.

## References

[B1] Aarsland D. Batzu L. Halliday G. M. Geurtsen G. J. Ballard C. Ray Chaudhuri K. (2021). Parkinson disease-associated cognitive impairment. *Nat. Rev. Dis. Primer* 7:47. 10.1038/s41572-021-00280-3 34210995

[B2] Abrevaya S. Sedeño L. Fitipaldi S. Pineda D. Lopera F. Buritica O. (2016). The road less traveled: Alternative pathways for action-verb processing in Parkinson’s disease. *J. Alzheimers Dis.* 55 1429–1435. 10.3233/JAD-160737 27834777

[B3] Angwin A. J. Chenery H. J. Copland D. A. Murdoch B. E. Silburn P. A. (2007). The speed of lexical activation is altered in Parkinson’s disease. *J. Clin. Exp. Neuropsychol.* 29 73–85. 10.1080/13803390500507188 17162724

[B4] Angwin A. J. Chenery H. J. Copland D. A. Murdoch B. E. Silburn P. A. (2005). Summation of semantic priming and complex sentence comprehension in Parkinson’s disease. *Cogn. Brain Res.* 25 78–89. 10.1016/j.cogbrainres.2005.04.008 15894470

[B5] Ash S. Xie S. X. Gross R. G. Dreyfuss M. Boller A. Camp E. (2012). The organization and anatomy of narrative comprehension and expression in Lewy body spectrum disorders. *Neuropsychology* 26 368–384. 10.1037/a0027115 22309984 PMC3348419

[B6] Brabenec L. Klobusiakova P. Barton M. Mekyska J. Galaz Z. Zvoncak V. (2019). Non-invasive stimulation of the auditory feedback area for improved articulation in Parkinson’s disease. *Parkinsonism Relat. Disord.* 61 187–192. 10.1016/j.parkreldis.2018.10.011 30337204

[B7] Brabenec L. Klobusiakova P. Simko P. Kostalova M. Mekyska J. Rektorova I. (2021). Non-invasive brain stimulation for speech in Parkinson’s disease: A randomized controlled trial. *Brain Stimulat.* 14 571–578. 10.1016/j.brs.2021.03.010 33781956

[B8] Colman K. S. F. Koerts J. Stowe L. A. Leenders K. L. Bastiaanse R. (2011). Sentence comprehension and its association with executive functions in patients with Parkinson’s disease. *Parkinsons Dis.* 2011 1–15. 10.4061/2011/213983 22135760 PMC3202107

[B9] Cramer S. C. Sur M. Dobkin B. H. O’Brien C. Sanger T. D. Trojanowski J. Q. (2011). Harnessing neuroplasticity for clinical applications. *Brain* 134 1591–1609. 10.1093/brain/awr039 21482550 PMC3102236

[B10] Dickerson B. C. Salat D. H. Greve D. N. Chua E. F. Rand-Giovannetti E. Rentz D. M. (2005). Increased hippocampal activation in mild cognitive impairment compared to normal aging and AD. *Neurology* 65 404–411. 10.1212/01.wnl.0000171450.97464.49 16087905 PMC4335677

[B11] Gajdoš M. Mikl M. Mareèek R. (2016). Mask_explorer: A tool for exploring brain masks in fMRI group analysis. *Comput. Methods Programs Biomed.* 134 155–163. 10.1016/j.cmpb.2016.07.015 27480740

[B12] Gross R. G. Camp E. McMillan C. T. Dreyfuss M. Gunawardena D. Cook P. A. (2013). Impairment of script comprehension in Lewy body spectrum disorders. *Brain Lang.* 125 330–343. 10.1016/j.bandl.2013.02.006 23566691 PMC3940934

[B13] Gross R. G. McMillan C. T. Chandrasekaran K. Dreyfuss M. Ash S. Avants B. (2012). Sentence processing in Lewy body spectrum disorder: The role of working memory. *Brain Cogn.* 78 85–93. 10.1016/j.bandc.2011.12.004 22218297 PMC3265703

[B14] Grossman M. Cooke A. DeVita C. Lee C. Alsop D. Detre J. (2003). Grammatical and resource components of sentence processing in Parkinson’s disease: An fMRI study. *Neurology* 60 775–781. 10.1212/01.WNL.0000044398.73241.13 12629232

[B15] Grossman M. Glosser G. Kalmanson J. Morris J. Stern M. B. Hurtig H. I. (2001). Dopamine supports sentence comprehension in Parkinson’s disease. *J. Neurol. Sci.* 184 123–130. 10.1016/S0022-510X(00)00491-3 11239945

[B16] Grossman M. Gross R. G. Moore P. Dreyfuss M. McMillan C. T. Cook P. A. (2012). Difficulty processing temporary syntactic ambiguities in Lewy body spectrum disorder. *Brain Lang.* 120 52–60. 10.1016/j.bandl.2011.08.007 21962945 PMC3253921

[B17] Grossman M. Irwin D. J. Jester C. Halpin A. Ash S. Rascovsky K. (2017). Narrative organization deficit in lewy body disorders is related to Alzheimer pathology. *Front. Neurosci.* 11:53. 10.3389/fnins.2017.00053 28228714 PMC5296303

[B18] Grossman M. Kalmanson J. Bernhardt N. Morris J. Stern M. B. Hurtig H. I. (2000). Cognitive resource limitations during sentence comprehension in Parkinson’s disease. *Brain Lang.* 73 1–16. 10.1006/brln.2000.2290 10872635

[B19] Grossman M. Lee C. Morris J. Stern M. B. Hurtig H. I. (2002). Assessing resource demands during sentence processing in Parkinson’s disease. *Brain Lang.* 80 603–616. 10.1006/brln.2001.2630 11896660

[B20] Hochstadt J. (2009). Set-shifting and the on-line processing of relative clauses in Parkinson’s disease: Results from a novel eye-tracking method. *Cortex* 45 991–1011. 10.1016/j.cortex.2009.03.010 19473654

[B21] Hochstadt J. Nakano H. Lieberman P. Friedman J. (2006). The roles of sequencing and verbal working memory in sentence comprehension deficits in Parkinson’s disease. *Brain Lang.* 97 243–257. 10.1016/j.bandl.2005.10.011 16332387

[B22] Hope T. R. Selnes P. Rektorová I. Anderkova L. Nemcova-Elfmarkova N. Balážová Z. (2019). Diffusion tensor and restriction spectrum imaging reflect different aspects of neurodegeneration in Parkinson’s disease. *PLoS One* 14:e0217922. 10.1371/journal.pone.0217922 31150514 PMC6544302

[B23] Johari K. Walenski M. Reifegerste J. Ashrafi F. Behroozmand R. Daemi M. (2019). A dissociation between syntactic and lexical processing in Parkinson’s disease. *J. Neurolinguistics* 51 221–235. 10.1016/j.jneuroling.2019.03.004 31777416 PMC6880793

[B24] Klobusiakova P. Mekyska J. Brabenec L. Galaz Z. Zvoncak V. Mucha J. (2021). Articulatory network reorganization in Parkinson’s disease as assessed by multimodal MRI and acoustic measures. *Parkinsonism Relat. Disord.* 84 122–128. 10.1016/j.parkreldis.2021.02.012 33609963

[B25] Kojovic M. Kassavetis P. Bologna M. Pareés I. Rubio-Agusti I. Beraredelli A. (2015). Transcranial magnetic stimulation follow-up study in early Parkinson’s disease: A decline in compensation with disease progression?: DECLINE in compensatory changes with PD progression. *Mov. Disord.* 30 1098–1106. 10.1002/mds.26167 25753906

[B26] Kotz S. A. Schwartze M. Schmidt-Kassow M. (2009). Non-motor basal ganglia functions: A review and proposal for a model of sensory predictability in auditory language perception. *Cortex* 45 982–990. 10.1016/j.cortex.2009.02.010 19361785

[B27] Lee C. Grossman M. Morris J. Stern M. B. Hurtig H. I. (2003). Attentional resource and processing speed limitations during sentence processing in Parkinson’s disease. *Brain Lang.* 85 347–356. 10.1016/S0093-934X(03)00063-4 12744946

[B28] Lowit A. Thies T. Steffen J. Scheele F. Roheger M. Kalbe E. (2022). Task-based profiles of language impairment and their relationship to cognitive dysfunction in Parkinson’s disease. *PLoS One* 17:e0276218. 10.1371/journal.pone.0276218 36301842 PMC9612451

[B29] Lucza T. Karádi K. Kállai J. Weintraut R. Janszky J. Makkos A. (2015). Screening mild and major neurocognitive disorders in Parkinson’s disease. *Behav. Neurol.* 2015:983606. 10.1155/2015/983606 26078489 PMC4452352

[B30] Marková J. Hajdúk M. Košutzká Z. Kušnírová A. Pápayová M. Egryová M. (2018). Sentence comprehension in Slovak-speaking patients with Parkinson disease. *Èes. Slov. Neurol. Neurochir.* 81 60–65. 10.14735/amcsnn201860

[B31] Marková J. Horváthová Ĺ Králová M. Cséfalvay Z. (2017). Sentence comprehension in Slovak-speaking patients with Alzheimer’s disease: Sentence comprehension in Alzheimer’s disease. *Int. J. Lang. Commun. Disord.* 52 456–468. 10.1111/1460-6984.12284 28000389

[B32] McGregor M. M. Nelson A. B. (2019). Circuit mechanisms of Parkinson’s disease. *Neuron* 101 1042–1056. 10.1016/j.neuron.2019.03.004 30897356

[B33] Mesulam M.-M. (1999). Neuroplasticity failure in Alzheimer’s disease. *Neuron* 24 521–529. 10.1016/S0896-6273(00)81109-5 10595506

[B34] Novakova L. Gajdos M. Rektorova I. (2020). Theta-burst transcranial magnetic stimulation induced cognitive task-related decrease in activity of default mode network: An exploratory study. *Brain Stimulat.* 13 597–599. 10.1016/j.brs.2020.01.015 32289683

[B35] O’Reilly J. X. Woolrich M. W. Behrens T. E. J. Smith S. M. Johansen-Berg H. (2012). Tools of the trade: Psychophysiological interactions and functional connectivity. *Soc. Cogn. Affect. Neurosci.* 7 604–609. 10.1093/scan/nss055 22569188 PMC3375893

[B36] Péran P. Cardebat D. Cherubini A. Piras F. Luccichenti G. Peppe A. (2009). Object naming and action-verb generation in Parkinson’s disease: A fMRI study. *Cortex* 45 960–971. 10.1016/j.cortex.2009.02.019 19368905

[B37] Poser B. A. Versluis M. J. Hoogduin J. M. Norris D. G. (2006). BOLD contrast sensitivity enhancement and artifact reduction with multiecho EPI: Parallel-acquired inhomogeneity-desensitized fMRI. *Magn. Reson. Med.* 55 1227–1235. 10.1002/mrm.20900 16680688

[B38] Postuma R. B. Berg D. Stern M. Poewe W. Olanow C. W. Oertel W. (2015). MDS clinical diagnostic criteria for Parkinson’s disease: MDS-PD Clinical Diagnostic Criteria. *Mov. Disord.* 30 1591–1601. 10.1002/mds.26424 26474316

[B39] Sejnoha Minsterova A. Klobusiakova P. Pies A. Galaz Z. Mekyska J. Novakova L. (2020). Patterns of diffusion kurtosis changes in Parkinson’s disease subtypes. *Parkinsonism Relat. Disord.* 81 96–102. 10.1016/j.parkreldis.2020.10.032 33120076

[B40] Skeel R. L. Crosson B. Nadeau S. E. Algina J. Bauer R. M. Fennell E. B. (2001). Basal ganglia dysfunction, working memory, and sentence comprehension in patients with Parkinson’s disease. *Neuropsychologia* 39 962–971. 10.1016/S0028-3932(01)00026-4 11516448

[B41] Smith K. M. Caplan D. N. (2018). Communication impairment in Parkinson’s disease: Impact of motor and cognitive symptoms on speech and language. *Brain Lang.* 185 38–46. 10.1016/j.bandl.2018.08.002 30092448

[B42] Troche M. S. Altmann L. J. P. (2012). Sentence production in Parkinson disease: Effects of conceptual and task complexity. *Appl. Psycholinguist.* 33 225–251. 10.1017/S0142716411000336

[B43] Tziortzi A. C. Searle G. E. Tzimopoulou S. Salinas C. Beaver J. D. Jenkinson M. (2011). Imaging dopamine receptors in humans with [11C]-(+)-PHNO: Dissection of D3 signal and anatomy. *NeuroImage* 54 264–277. 10.1016/j.neuroimage.2010.06.044 20600980

[B44] Walenski M. Europa E. Caplan D. Thompson C. K. (2019). Neural networks for sentence comprehension and production: An ALE-based meta-analysis of neuroimaging studies. *Hum. Brain Mapp.* 40 2275–2304. 10.1002/hbm.24523 30689268 PMC6597252

[B45] Ye Z. Milenkova M. Mohammadi B. Kollewe K. Schrader C. Dengler R. (2012). Impaired comprehension of temporal connectives in Parkinson’s disease—A neuroimaging study. *Neuropsychologia* 50 1794–1800. 10.1016/j.neuropsychologia.2012.04.004 22561179

