# Supplementary material for: Language impairment in Parkinson’s disease: fMRI study of sentence reading comprehension
Source: Front Aging Neurosci. 2023 Mar 9;15:1117473. doi: 10.3389/fnagi.2023.1117473 (PMC10033839; doi:10.3389/fnagi.2023.1117473)
Supplement: Supplementary file 1 [file Data_Sheet_1.docx]

**Supplementary materials**

**Test of Sentence Comprehension, sentences description:**

All tested sentences were active and all of them were unambiguous because of the morphological cue at nouns and pronouns. Canonical sentences were subject-verb-object (SVO) sentences and a centre-embedded relative clause with a relative pronoun substituting for subject (ES) sentences, non-canonical were object-verb-subject (OVS) sentences and a centre-embedded relative clause with a relative pronoun substituting for object (EO) sentences. The examples are below:

**SVO:**

Mam**a-NOM** so svetlými vlasmi umýva chlapc**a-ACC**.

Mother-**NOM** with bright hair is washing boy-**ACC.**

**OVS:**

Chlapc**a-ACC** umýva mam**a**-**NOM** so svetlými vlasmi.

Boy-**ACC** is washing mother-**NOM** with bright hair.

**ES:**

Chlap**ec-NOM**, ktor**ý-NOM** umýva mam**u**-**ACC**, má tmavé vlasy.

Boy-**NOM**, which-**NOM** is washing mother-**ACC**, has dark hair.

**EO:**

Chlap**ec-NOM**, kter**ého-ACC** umýva mam**a-NOM**, má svetlé vlasy.

Boy-**NOM**, which-**ACC** is washing mother-**NOM,** has bright hair.

From listed syntactic structures only OVS sentences are typical for Slovak language and do not exist in English. Complex ES a EO sentences have similar structure as sentences in English. Behavioral experiments of many languages with flexible word orders have repeatedly reported that canonical word order has a processing advantage over non-canonical word order. One possible factor is conceptual accessibility, the ease with which the mental representation of some potential referent can be activated in or retrieved from memory (Tamaoka et al. 2005).

Tamaoka, K., Sakai, H., Kawahara, J., Miyaoka, Y., Lim, H., & Koizumi, M. (2005). Priority information used for the processing of Japanese sentences: Thematic roles, case particles or grammatical functions? Journal of Psycholinguistic Research, 34, 281–332

|  | Spearman r | Spearman p |
| --- | --- | --- |
| Correlation of Accuracy_noncanonical with MoCA | 0.14 | 0.55 |
| Correlation of Accuracy_noncanonical with Rey copy | 0.29 | 0.21 |
| Correlation of Accuracy_noncanonical with Rey3 | 0.05 | 0.82 |
| Correlation of Accuracy_noncanonical with Rey30 | 0.16 | 0.50 |
| Correlation of Accuracy_noncanonical with AVLT 1 to 5 | 0.01 | 0.98 |
| Correlation of Accuracy_noncanonical with ALVT6 | -0.11 | 0.65 |
| Correlation of Accuracy_noncanonical with AVLT30 | -0.09 | 0.72 |
| Correlation of Accuracy_noncanonical with stroop_W | 0.01 | 0.98 |
| Correlation of Accuracy_noncanonical with stroop_C | -0.07 | 0.77 |
| Correlation of Accuracy_noncanonical with stroop_CW | 0.28 | 0.23 |
| Correlation of Accuracy_noncanonical with stroop_IF | 0.43 | 0.06 |
| Correlation of Accuracy_noncanonical with TMTA | 0.27 | 0.24 |
| Correlation of Accuracy_noncanonical with TMTB | -0.21 | 0.38 |
| Correlation of Accuracy_noncanonical with repetition of numbers and letters | 0.11 | 0.65 |
| Correlation of Accuracy_noncanonical with semantic fluency | 0.11 | 0.65 |
| Correlation of Accuracy_noncanonical with phonemic fluency | 0.40 | 0.08 |
| Correlation of Accuracy_all_task with MoCA | 0.22 | 0.35 |
| Correlation of Accuracy_all_task with Rey copy | 0.32 | 0.17 |
| Correlation of Accuracy_all_task with Rey3 | 0.13 | 0.59 |
| Correlation of Accuracy_all_task with Rey30 | 0.21 | 0.36 |
| Correlation of Accuracy_all_task with AVLT 1 to 5 | 0.09 | 0.70 |
| Correlation of Accuracy_all_task with ALVT6 | 0.02 | 0.94 |
| Correlation of Accuracy_all_task with AVLT30 | 0.07 | 0.76 |
| Correlation of Accuracy_all_task with stroop_W | 0.12 | 0.63 |
| Correlation of Accuracy_all_task with stroop_C | 0.01 | 0.97 |
| Correlation of Accuracy_all_task with stroop_CW | 0.28 | 0.23 |
| Correlation of Accuracy_all_task with stroop_IF | 0.38 | 0.10 |
| Correlation of Accuracy_all_task with TMTA | 0.30 | 0.20 |
| Correlation of Accuracy_all_task with TMTB | -0.30 | 0.21 |
| Correlation of Accuracy_all_task with repetition of numbers and letters | 0.21 | 0.38 |
| Correlation of Accuracy_all_task with semantic fluency | 0.26 | 0.27 |
| Correlation of Accuracy_all_task with phonemic fluency | 0.49 | 0.03 |

Table S1: Correlation of neuropsychological measures with Test of sentence comprehension (ToSC) task accuracy. Medium to strong correlations with |r|>0.4 or p<0.05 are highlighted in red. Legend: Montreal Cognitive Assessment (MOCA), Rey’s figure- (copy, immediate – Rey3 a delayed recall – Rey30), auditory verbal learning test (AVLT) —  memory learning test (immediate – AVLT1 to 5, after interference – AVLT6 and delayed verbal memory- AVLT 30), Stroop test W (words), C (colours), CW (colour-word), IF (inteference), Trail Making Test —  processing speed (Part A - TMTA), cognitive flexibility and task switching (Part B -TMTB)

| **HC one sample t-test right striatum** | | | |  |  |  |  |
| --- | --- | --- | --- | --- | --- | --- | --- |
|  |  |  |  |  |  |  |  |
| cluster | cluster | voxel |  |  |  |  |  |
| p(cor) | equivk | T | x,y,z {mm} | | | AAL label |  |
| 0.00E+00 | 2469 | 10.22 | -19.32 | 13.49 | -7.50 | Putamen L | |
|  |  | 8.84 | -9.07 | 3.25 | 2.50 | Pallidum L | |
| 6.57E-06 | 231 | 7.82 | 16.54 | -19.80 | 70.00 | Precentral R | |
|  |  | 6.12 | 26.78 | -17.24 | 67.50 | Precentral R | |
|  |  | 5.72 | 6.29 | -9.56 | 67.50 | Supp Motor Area R | |
| 8.09E-07 | 276 | 7.63 | 57.51 | -42.85 | -15.00 | Temporal Inf R | |
|  |  | 6.03 | 49.83 | -19.80 | -15.00 | Temporal Mid R | |
|  |  | 5.84 | 65.19 | -32.61 | -15.00 | Temporal Mid R | |
| 8.37E-09 | 383 | 7.58 | 42.15 | 18.61 | 27.50 | Frontal Inf Tri R | |
|  |  | 6.76 | 52.39 | 0.68 | 35.00 | Precentral R | |
|  |  | 6.22 | 37.02 | 8.37 | 30.00 | Frontal Inf Oper R | |
| 5.69E-06 | 234 | 6.33 | -60.29 | -45.41 | -12.50 | Temporal Mid L | |
|  |  | 5.96 | -60.29 | -35.17 | -17.50 | Temporal Inf L | |
|  |  | 5.22 | -62.86 | -24.93 | -22.50 | Temporal Inf L | |
| 1.66E-05 | 212 | 5.99 | 21.66 | -86.39 | -5.00 | Lingual R |  |
|  |  | 5.24 | 13.97 | -81.27 | -10.00 | Lingual R |  |
|  |  | 4.65 | 34.46 | -83.83 | -15.00 | Occipital Inf R | |
| 9.25E-04 | 136 | 5.92 | 11.41 | 16.05 | 32.50 | Cingulum Mid R | |
|  |  | 5.04 | 6.29 | 18.61 | 40.00 | Cingulum Mid R | |
| 7.12E-09 | 387 | 5.85 | -6.51 | -68.46 | 40.00 | Precuneus L | |
|  |  | 5.71 | 3.73 | -73.58 | 35.00 | Precuneus R | |
|  |  | 5.35 | 34.46 | -73.58 | 42.50 | Occipital Sup R | |
| 4.13E-07 | 291 | 5.81 | -42.37 | 8.37 | 25.00 | Frontal Inf Oper L | |
|  |  | 5.68 | -34.68 | 5.81 | 25.00 | Frontal Inf Oper L | |
|  |  | 5.53 | -50.05 | 13.49 | 30.00 | Frontal Inf Tri L | |
| 6.39E-08 | 334 | 5.41 | -57.73 | -58.22 | 7.50 | Temporal Mid L | |
|  |  | 5.40 | -55.17 | -45.41 | 10.00 | Temporal Mid L | |
|  |  | 4.38 | -42.37 | -58.22 | 17.50 | Temporal Mid L | |
| 8.86E-07 | 274 | 5.30 | -6.51 | -65.90 | 7.50 | Lingual L |  |
|  |  | 5.24 | -27.00 | -81.27 | -15.00 | Lingual L |  |
|  |  | 4.53 | 6.29 | -68.46 | 5.00 | Lingual R |  |
| 3.02E-04 | 156 | -2.98 | 37.02 | 3.25 | 5.00 | Insula R |  |
|  |  | -2.98 | 34.46 | 5.81 | -2.50 | Putamen R | |
|  |  |  |  |  |  |  |  |
| **PD one sample t-test right striatum** | | | |  |  |  |  |
|  |  |  |  |  |  |  |  |
| cluster | cluster | voxel |  |  |  |  |  |
| p(cor) | equivk | T | x,y,z {mm} | | | AAL label |  |
| 0.00E+00 | 2025 | 8.20 | 8.85 | -63.34 | 50.00 | Precuneus R | |
|  |  | 7.53 | -3.95 | -50.54 | 52.50 | Precuneus L | |
| 5.69E-12 | 511 | 7.50 | -14.20 | 16.05 | 0.00 | Caudate L | |
|  |  | 6.77 | 6.29 | -9.56 | 12.50 | Thalamus R | |
|  |  | 6.32 | 3.73 | -1.88 | 5.00 | Out of atlas | |
| 1.92E-06 | 229 | 7.08 | -50.05 | -58.22 | -17.50 | Temporal Inf L | |
|  |  | 5.44 | -57.73 | -42.85 | -15.00 | Temporal Mid L | |
|  |  | 5.10 | -50.05 | -58.22 | -7.50 | Temporal Inf L | |
| 0.00E+00 | 1112 | 6.94 | 6.29 | 16.05 | 57.50 | Supp Motor Area R | |
|  |  | 6.62 | -6.51 | -12.12 | 67.50 | Paracentral Lobule L | |
|  |  | 6.56 | -9.07 | -19.80 | 47.50 | Cingulum Mid L | |
| 2.46E-03 | 107 | 6.82 | 34.46 | -50.54 | -32.50 | Cerebellum 6 R | |
|  |  | 5.49 | 34.46 | -60.78 | -32.50 | Cerebellum Crus1 R | |
|  |  | 3.87 | 47.27 | -53.10 | -25.00 | Temporal Inf R | |
| 5.57E-10 | 401 | 6.77 | 39.58 | 16.05 | 25.00 | Frontal Inf Tri R | |
|  |  | 6.26 | 54.95 | 0.68 | 37.50 | Precentral R | |
|  |  | 5.85 | 34.46 | 21.17 | 32.50 | Frontal Mid R | |
| 4.79E-07 | 256 | 6.59 | -34.68 | -60.78 | -47.50 | Cerebellum 8 L | |
|  |  | 5.69 | -42.37 | -37.73 | -27.50 | Temporal Inf L | |
|  |  | 5.57 | -39.81 | -53.10 | -32.50 | Cerebellum Crus1 L | |
| 5.23E-03 | 96 | 6.26 | 8.85 | 16.05 | 37.50 | Cingulum Mid R | |
|  |  | 4.78 | 6.29 | 46.78 | 37.50 | Frontal Sup Medial R | |
|  |  | 4.54 | 11.41 | 33.98 | 35.00 | Frontal Sup Medial R | |
| 3.32E-10 | 413 | 6.04 | -42.37 | -7.00 | 35.00 | Precentral L | |
|  |  | 5.19 | -39.81 | 10.93 | 25.00 | Frontal Inf Tri L | |
|  |  | 5.14 | -32.12 | 26.29 | 27.50 | Frontal Mid L | |
| 3.23E-02 | 71 | 5.76 | 34.46 | -32.61 | -20.00 | Fusiform R | |
|  |  | 5.02 | 37.02 | -19.80 | -25.00 | Parahippocampal R | |
| 2.39E-02 | 75 | 4.48 | -9.07 | -40.29 | 2.50 | Precuneus L | |
|  |  | 4.05 | 6.29 | -40.29 | 5.00 | Cingulum Post R | |
|  |  | 4.02 | 6.29 | -42.85 | 15.00 | Cingulum Post R | |
|  |  |  |  |  |  |  |  |
| **HC one sample t-test left striatum** | | | |  |  |  |  |
|  |  |  |  |  |  |  |  |
| cluster | cluster | voxel |  |  |  |  |  |
| p(cor) | equivk | T | x,y,z {mm} | | | AAL label |  |
| 0.00E+00 | 2221 | 10.23 | 13.97 | 18.61 | -7.50 | Caudate R | |
|  |  | 8.81 | 11.41 | 13.49 | 0.00 | Caudate R | |
| 0.00E+00 | 1599 | 7.46 | 57.51 | -37.73 | -15.00 | Temporal Mid R | |
|  |  | 7.29 | 29.34 | -19.80 | -22.50 | Parahippocampal R | |
|  |  | 6.65 | 31.90 | -71.02 | -15.00 | Fusiform R | |
| 0.00E+00 | 2654 | 7.23 | -11.64 | -68.46 | 50.00 | Precuneus L | |
|  |  | 6.97 | -37.25 | -86.39 | 5.00 | Occipital Mid L | |
|  |  | 6.90 | 29.34 | -68.46 | 50.00 | Parietal Sup R | |
| 3.55E-02 | 81 | 5.78 | 54.95 | -55.66 | 15.00 | Temporal Mid R | |
|  |  | 4.92 | 52.39 | -53.10 | 22.50 | Temporal Sup R | |
|  |  | 3.50 | 42.15 | -60.78 | 22.50 | Angular R |  |
| 5.49E-05 | 198 | 5.47 | 42.15 | 21.17 | 27.50 | Frontal Inf Tri R | |
|  |  | 5.25 | 34.46 | 3.25 | 32.50 | Precentral R | |
|  |  | 4.62 | 52.39 | -1.88 | 37.50 | Precentral R | |
| 7.50E-08 | 348 | 5.27 | -47.49 | -19.80 | -10.00 | Temporal Mid L | |
|  |  | 5.24 | -62.86 | -35.17 | -15.00 | Temporal Mid L | |
|  |  | 5.22 | -62.86 | -42.85 | -12.50 | Temporal Mid L | |
| 4.03E-02 | 79 | 4.54 | 19.10 | -68.46 | -50.00 | Cerebellum 8 R | |
|  |  | 4.52 | 26.78 | -83.83 | -37.50 | Cerebellum Crus2 R | |
|  |  | 4.22 | 16.54 | -81.27 | -42.50 | Cerebellum Crus2 R | |
|  |  |  |  |  |  |  |  |
| **PD one sample t-test left striatum** | | | |  |  |  |  |
|  |  |  |  |  |  |  |  |
| cluster | cluster | voxel |  |  |  |  |  |
| p(cor) | equivk | T | x,y,z {mm} | | | AAL label |  |
| 0.00E+00 | 3985 | 9.80 | 1.17 | -53.10 | 60.00 | Precuneus R | |
|  |  | 8.42 | 6.29 | 31.42 | 60.00 | Frontal Sup Medial R | |
|  |  | 8.29 | -3.95 | -50.54 | 52.50 | Precuneus L | |
| 1.05E-13 | 585 | 9.30 | 11.41 | 3.25 | 10.00 | Caudate R | |
| 3.26E-02 | 68 | 8.02 | 26.78 | -30.05 | 65.00 | Precentral R | |
|  |  | 5.40 | 34.46 | -27.49 | 62.50 | Precentral R | |
| 0.00E+00 | 1548 | 7.80 | -52.61 | -35.17 | -25.00 | Temporal Inf L | |
|  |  | 7.58 | -37.25 | -60.78 | -15.00 | Fusiform L | |
|  |  | 7.36 | -27.00 | -76.15 | -40.00 | Cerebellum Crus2 L | |
| 0.00E+00 | 1006 | 7.69 | -44.93 | 41.66 | -12.50 | Frontal Inf Orb L | |
|  |  | 7.37 | -52.61 | -1.88 | 45.00 | Precentral L | |
|  |  | 7.27 | -37.25 | 49.34 | 0.00 | Frontal Mid L | |
| 1.18E-09 | 366 | 6.47 | 13.97 | -76.15 | -22.50 | Cerebellum 6 R | |
|  |  | 6.11 | 29.34 | -71.02 | -25.00 | Cerebellum 6 R | |
|  |  | 5.64 | 31.90 | -45.41 | -32.50 | Cerebellum 6 R | |
| 1.53E-05 | 182 | 5.19 | 37.02 | -83.83 | 2.50 | Occipital Mid R | |
|  |  | 4.37 | 24.22 | -94.07 | 0.00 | Calcarine R | |
| 3.04E-07 | 253 | 6.11 | 11.41 | -63.34 | 7.50 | Calcarine R | |
|  |  | 5.48 | 1.17 | -50.54 | 5.00 | Vermis 4 5 | |
|  |  | 5.23 | -6.51 | -35.17 | -5.00 | Cerebellum 4 5 L | |
| 5.21E-03 | 92 | 6.05 | -9.07 | 57.03 | -12.50 | Frontal Med Orb L | |
|  |  | 5.07 | -9.07 | 54.47 | 2.50 | Cingulum Ant L | |
|  |  | 4.51 | -1.39 | 54.47 | -2.50 | Cingulum Ant L | |
| 4.15E-02 | 65 | 5.94 | -6.51 | 51.90 | 32.50 | Frontal Sup Medial L | |
|  |  | 5.65 | -16.76 | 54.47 | 30.00 | Frontal Sup L | |
|  |  | 4.11 | 3.73 | 57.03 | 32.50 | Frontal Sup Medial R | |
| 3.62E-03 | 97 | 5.84 | -32.12 | -73.58 | 27.50 | Occipital Mid L | |
|  |  | 4.93 | -24.44 | -76.15 | 30.00 | Occipital Mid L | |
| 1.03E-03 | 115 | 4.88 | 37.02 | -71.02 | -50.00 | Cerebellum 7b R | |
|  |  | 4.46 | 29.34 | -71.02 | -47.50 | Cerebellum 7b R | |
|  |  | 4.35 | 34.46 | -63.34 | -50.00 | Cerebellum 8 R | |
|  |  |  |  |  |  |  |  |

Table S2: PPI seed connectivity from the left or right striatum with significant regions for HC and PD with the figure depicted these regions below. We have depicted clusters significant on cluster level inference (p corrected <0.05) with initial cutoff of p = 0.005 present in grey matter. Each cluster is characterized by the size and parameters of its maxima (T value and position).


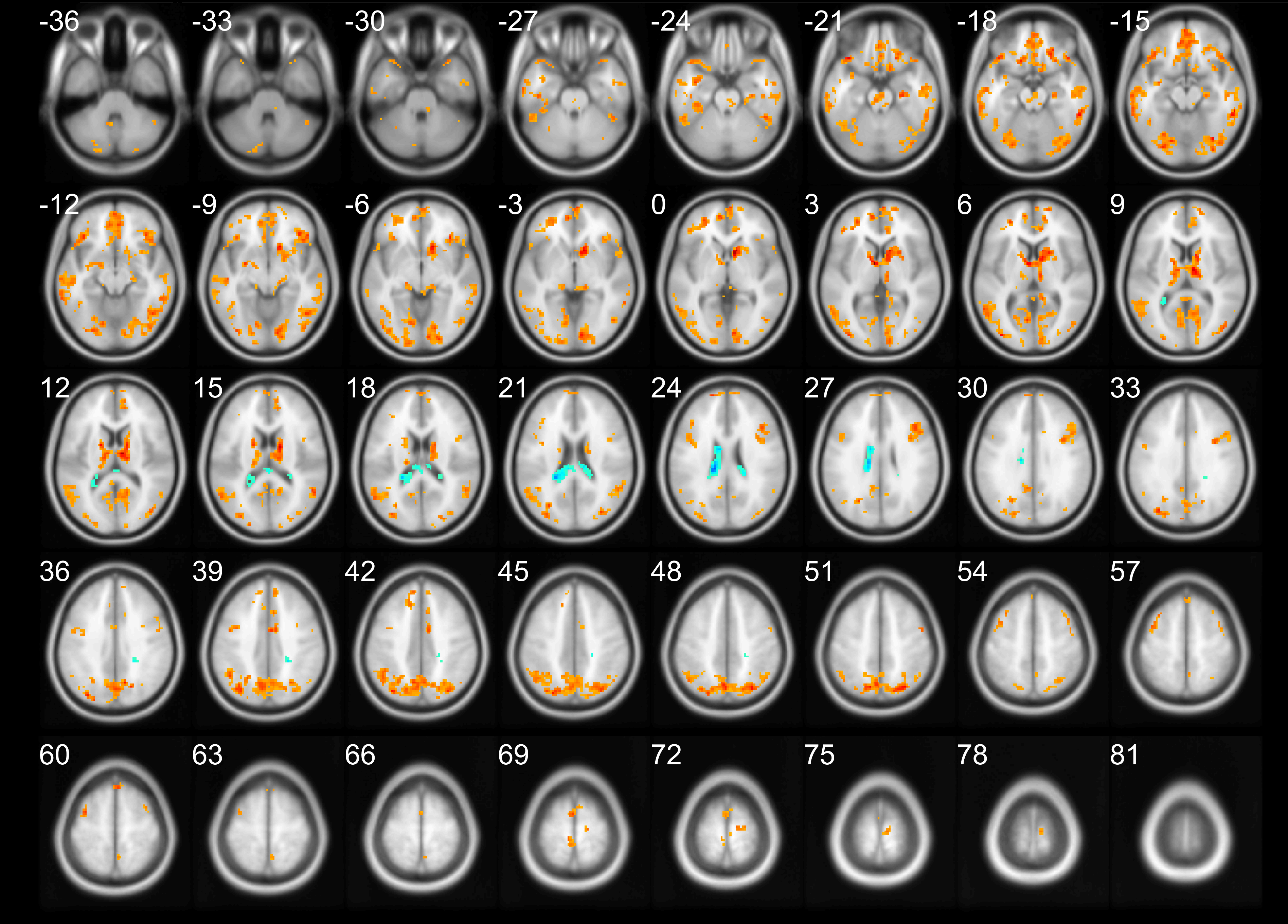


Figure S1: Slice view of one sample t-test for HC on seed PPI connectivity from left striatum for noncanonical stimuli. Only p < 0.005 are overlaid.


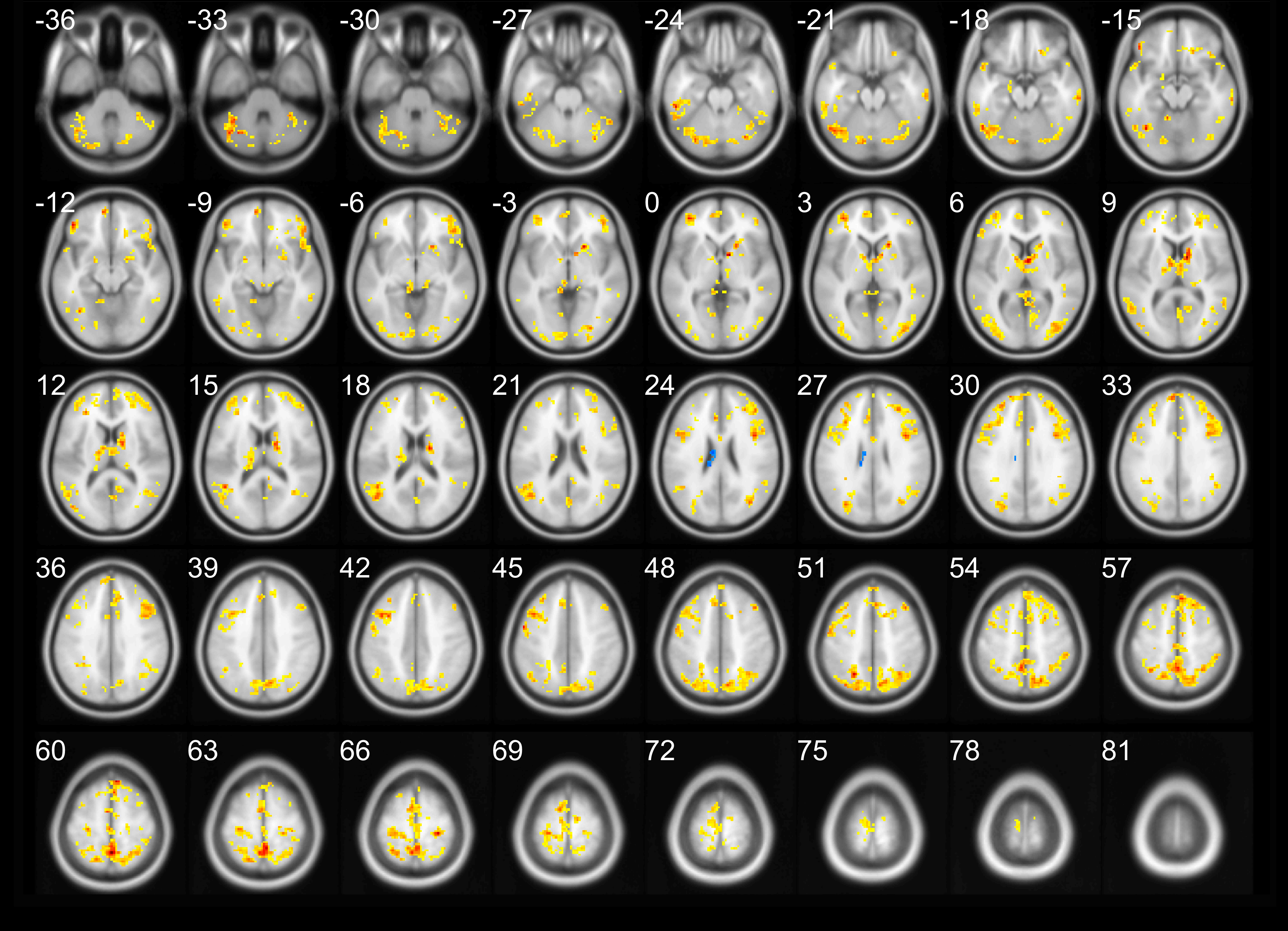


Figure S2: Slice view of one sample t-test for PD on seed PPI connectivity from left striatum for noncanonical stimuli. Only p < 0.005 are overlaid.


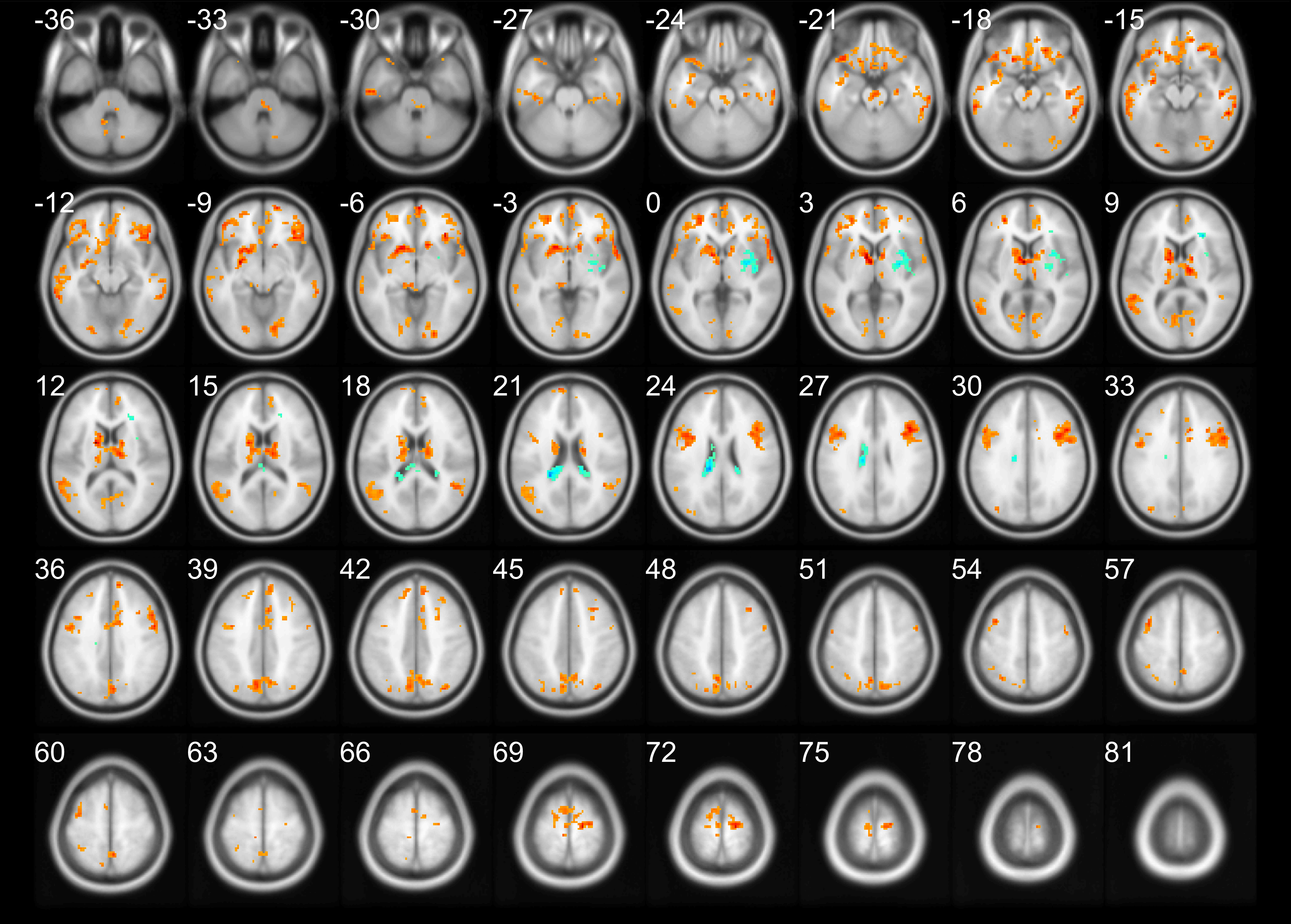


Figure S3: Slice view of one sample t-test for HC on seed PPI connectivity from right striatum for noncanonical stimuli. Only p < 0.005 are overlaid.


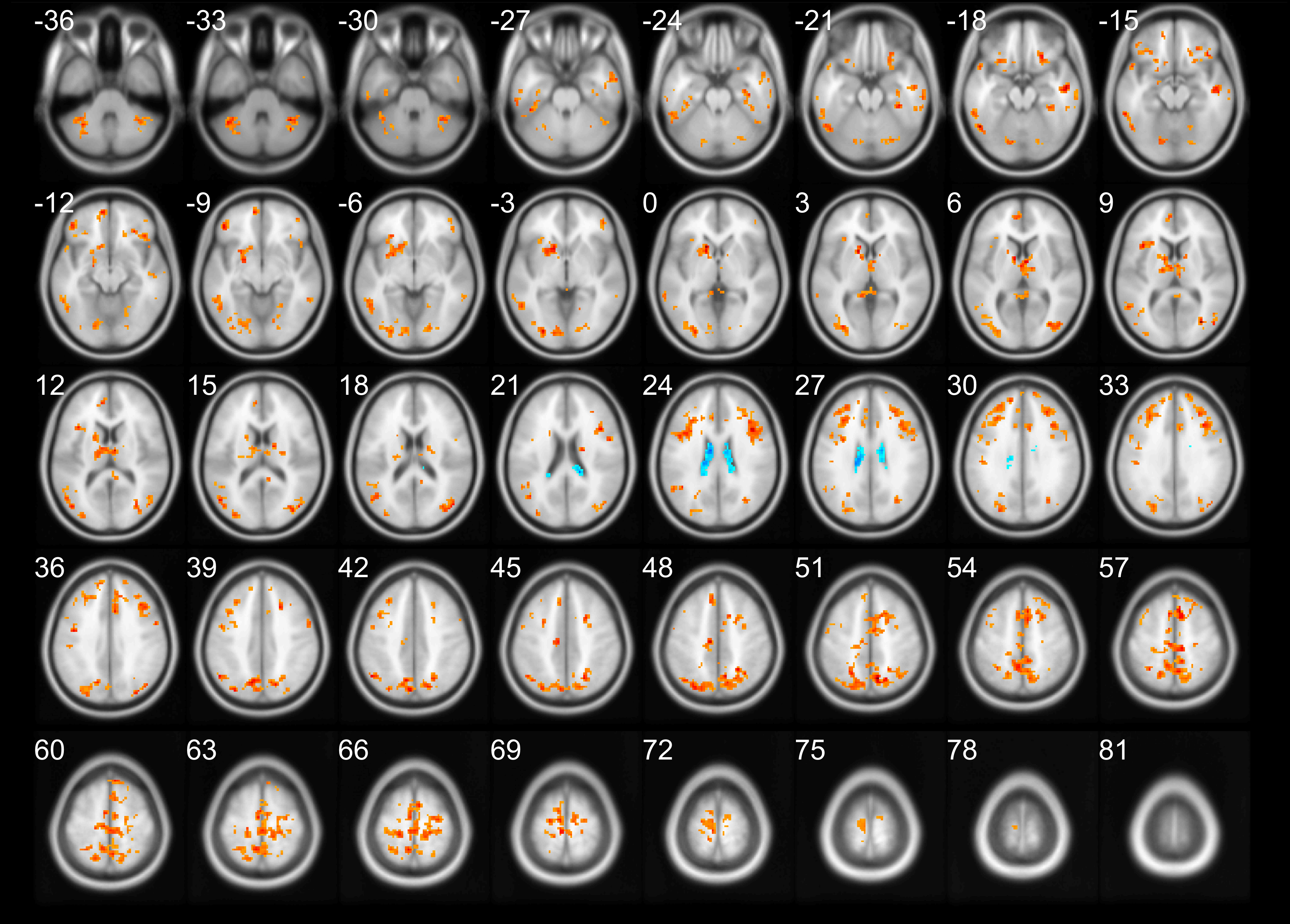


Figure S4: Slice view of one sample t-test for PD on seed PPI connectivity from right striatum for noncanonical stimuli. Only p < 0.005 are overlaid.
